# Cardiac Progenitor Cell–Derived Extracellular Vesicles Reduce Infarct Size and Associate with Increased Cardiovascular Cell Proliferation

**DOI:** 10.1007/s12265-018-9842-9

**Published:** 2018-11-19

**Authors:** Janita A. Maring, Kirsten Lodder, Emma Mol, Vera Verhage, Karien C. Wiesmeijer, Calinda K. E. Dingenouts, Asja T. Moerkamp, Janine C. Deddens, Pieter Vader, Anke M. Smits, Joost P. G. Sluijter, Marie-José Goumans

**Affiliations:** 1 Laboratory of Cardiovascular Cell Biology, Department of Cell and Chemical Biology, Leiden University Medical Centre, Leiden, The Netherlands; 2 Laboratory of Experimental Cardiology, University Medical Center Utrecht, University Utrecht, Utrecht, The Netherlands; 3 Department of Clinical Chemistry and Hematology, University Medical Center Utrecht, Utrecht, The Netherlands; 4 UMC Utrecht Regenerative Medicine Center, University Medical Center Utrecht, Utrecht, The Netherlands

**Keywords:** Extracellular vesicles, Cardiac progenitor cells, Myocardial infarction, Proliferation, Cardiomyocytes, Angiogenesis, Endoglin

## Abstract

Cell transplantation studies have shown that injection of progenitor cells can improve cardiac function after myocardial infarction (MI). Transplantation of human cardiac progenitor cells (hCPCs) results in an increased ejection fraction, but survival and integration are low. Therefore, paracrine factors including extracellular vesicles (EVs) are likely to contribute to the beneficial effects. We investigated the contribution of EVs by transplanting hCPCs with reduced EV secretion. Interestingly, these hCPCs were unable to reduce infarct size post-MI. Moreover, injection of hCPC-EVs did significantly reduce infarct size. Analysis of EV uptake showed cardiomyocytes and endothelial cells primarily positive and a higher Ki67 expression in these cell types. Yes-associated protein (YAP), a proliferation marker associated with Ki67, was also increased in the entire infarcted area. In summary, our data suggest that EV secretion is the driving force behind the short-term beneficial effect of hCPC transplantation on cardiac recovery after MI.

**Electronic supplementary material:**

The online version of this article (10.1007/s12265-018-9842-9) contains supplementary material, which is available to authorized users.

## Introduction

Cardiovascular disease is one of the most prevalent causes of mortality in the world, and myocardial infarction (MI) attributes to almost one third of its deaths [1]. Though many of the risk factors leading to MI can be treated to prevent recurrence, the tissue damage remains untreatable to date. The loss of cardiomyocytes is permanent, reducing cardiac output and ultimately leading to end-stage heart failure. To prevent heart failure, therapies aiming to reduce damage to the injured myocardial wall and/or replace the lost cardiomyocytes have been explored. One approach to repair cardiac tissue was to inject human heart–derived cardiac progenitor cells (hCPCs) into the border zone of the infarction. Since hCPCs are able to differentiate into cardiomyocytes, endothelial cells, and smooth muscle cells in vitro [2, 3], they are likely candidates to replenish the infarcted heart with new cardiovascular cells. Transplantation of hCPCs into the infarct border zone has indeed been shown to lead to a sustained stabilization in cardiac function up to 3 months post-MI and was accompanied by a significant increase in the number of murine vessels in the infarct border zone [4]. However, only a small percentage of the injected hCPCs remained in the mouse heart 3 months after transplantation [4]. Two weeks after transplantation, an increase in vessel density was already discerned, with no evidence of vascular differentiation yet of the injected progenitor cells [5]. Most interestingly, an effect on cardiac function was observed as early as 2 days post-MI [4]. These observations suggest that hCPCs positively influence the post-MI environment through the secretion of paracrine factors, instead of direct differentiation into cardiovascular cells that rebuild the myocardial wall. This is further supported by our observation that extracellular vesicles (EVs) isolated from hCPCs have a great pro-angiogenic potential in vitro and in vivo [6, 7], due to the presence of several pro-angiogenic factors in and on these EVs, of which extracellular matrix metalloproteinase inducer (EMMPRIN) is a major determinant [6, 7].

EVs form a population comprising a multitude of microvesicles, of which the smallest is termed exosomes, ranging from 50 to 150 nm in size. Exosomes have a lipid bilayer membrane and their content includes mRNAs, miRNAs, and proteins [8–10]. EVs are able to influence many aspects of cell behavior, such as migration and proliferation, modulate the immune response, and can signal over both short and long distances [6, 11–13]. These influential characteristics make EVs promising as therapy after MI, since after injection, they potentially can affect the cardiovascular cells in the damaged and ischemic area.

Another effect of hCPC transplantation is an increase in the number of PCNA-expressing cells in the mouse myocardium [5]. Inducing proliferation of the surviving endogenous cardiomyocytes can also lead to restoration of cardiac contractility. Unfortunately, although proliferation of cardiomyocytes is observed in neonates, this capacity reduces dramatically shortly after birth [14]. Until recently, the adult mammalian heart was considered a post-mitotic organ [15], but Bergmann et al. showed that cardiomyocyte turnover was present in the adult heart, albeit at only 0.3–1% per year [16, 17]. The transcription factor Meis1 has been shown to be involved in the decrease in cardiomyocyte proliferation after birth [18], while other studies have shown the involvement of the Hippo pathway in cardiomyocyte proliferation [19, 20]. Inhibition of Hippo pathway components leads to activation of YAP, which induces proliferation of cardiomyocytes [21, 22].

Since hCPC transplantation after MI improves cardiac function within 48 h after injection [4], which is too early to be the result of hCPC differentiation [5], hCPC transplantation does increase the number of PCNA-expressing cells in the mouse myocardium [5], and hCPCs secrete EVs that are pro-angiogenic [6, 7], the aim of this study is to determine if hCPC-secreted EVs are responsible for the early beneficial effects after hCPC transplantation.

## Materials and Methods

### Cell Culture

Primary hCPC isolation of human heart tissue was performed as previously described [23, 24]. Human heart tissue was obtained based on individual informed consent, after approval by the Medical Ethics committee of the Leiden University Medical Center. The investigation conforms with the principles outlined in the Declaration of Helsinki. For the isolation of EVs, hCPCs (fetal CPCs, HFH17.1) were cultured for 4 days in medium containing EV-depleted serum as described before [7]. Human microvascular endothelial cells (HMEC-1, ATCC), used for functional assessment of the hCPC-EV activity [6], were isolated as previously described [25, 26] and cultured on fibronectin-coated flasks in MCDB131 medium (without L-glutamine) with 10% fetal bovine serum (FBS), 10 ng/mL epidermal growth factor (EGF), 1 μg/mL hydrocortisone, 10 mM glutamine, and 1% penicillin/streptomycin. All cells were cultured at 37 °C with 5% CO2.

### EV Isolation and Labelling

After culturing hCPCs for 4 days, conditioned medium was harvested, and hCPC-EVs were isolated by differential centrifugation with a final step at 110,000×*g*, as described previously [7]. Density separation was performed by sucrose gradient. EVs were mixed into the bottom layer containing 2.5 M sucrose, followed by a layered gradient ranging from 2 to 0.4 M sucrose. The gradient was centrifuged for 16 h at 190,000×*g*, divided in 12 fractions, washed with phosphate-buffered saline (PBS), centrifuged 70 min at 110,000×*g*, and used for Western blot analysis. To label EVs, PKH26 or PKH67 (Sigma) was incubated with isolated EVs according to the manufacturer’s protocol. After a 10-min incubation, labelling was stopped by adding FBS (ultra-EV free, centrifuged overnight at 160,000×*g*), and EVs were isolated by a sucrose gradient as described above. The fraction containing EVs was washed with PBS and centrifuged for 70 min at 110,000×*g*. Pellet was resuspended in PBS and diluted to a concentration of 0.8 μg/μL based on EV protein, assessed by BCA assay (Thermo Fischer).

### Characterization of EVs

EV characterization was performed by loading 10 μL of EV-isolate, corrected for equal cell numbers, onto a 10% SDS-PAGE gel and Western blot analysis for flotillin-1 (rabbit, SC-25506, Santa Cruz), CD9 (sc-53,679, Santa Cruz), CD63 (CBL-553, Millipore), CD81, ALIX, TSG101, and EMMPRIN (10R-CD147alphaHu, Fitzgerald) [27]. Purity of EV isolates was confirmed by absence of calnexin (mouse, SC-80645, Santa Cruz) and underrepresentation of AGO. EV size was analyzed with qNano (Izon) to confirm size distribution and concentration, and TEM was used to visualize the vesicles. EV functionality was confirmed using a scratch assay [7]. A scratch was made in a monolayer of HMEC-1 cells and any floating cells were removed. Medium was replaced by basal MCDB131 with or without the addition of 5 μg/mL EVs (determined by BCA assay, Thermo Fischer), or equal volumes of EV-isolate (corrected for equal cell numbers). Percentage closure was determined after 24 h.

### Rab27A and Rab27B Knockdown

Knockdown of Rab27A and Rab27B was established via lentiviral-mediated shRNA (TRCN0000380306, TRCN0000294016, MISSION library, Sigma). As a control, cells were transduced with a shRNA targeting luciferase (SHC007, MISSION library). Medium was changed after 16 h and the cells were subjected to puromycin selection for 48 h. Knockdown was verified by qPCR. RNA was isolated using TriPure (Sigma), according to manufacturer’s specifications. cDNA was synthesized with the RevertAid First Strand cDNA Synthesis Kit (Thermo Scientific) using 1 μg of RNA. Expression levels of Rab27A and Rab27B were assessed by quantitative real-time PCR using SYBR-green mastermix (Bio-Rad Laboratories). Housekeeping genes GAPDH, β-actin, and ARP were used to normalize expression levels. For further EV analysis (Western Blot, qNano, scratch analysis), volumes of EV isolates were corrected for number of cells.

### Myocardial Infarction by Ligation of the Left Anterior Descending Artery

All animal experiments were approved by the Animal Ethical Experimentation Committee of the Leiden University Medical Center and carried out in accordance with the Guide for the Care and Use of Laboratory Animals. Male NOD-SCID mice, 10–12 weeks old, were anesthetized by intraperitoneal injection (i.p.) of 200 μL anesthesia mixture (0.05 mg/mL dexmedetomidine, 0.01 mg/mL fentanyl, and 1 mg/mL midazolam) and subsequently intubated. The left anterior descending artery (LAD) was permanently ligated as described previously [28]. After 15 min, PBS, 0.5*10^6^ hCPCs, or 8 μg of purified EVs were injected into the border zone of the infarction, with two injections of 5 μL on each side of the ligation. Injections were randomly assigned. The mice were awakened by i.p. injection of an antagonist mixture (100 μL, 0.08 mg/mL flumazenil, 0.4 mg/mL atipamezole, 0.025 mg/mL buprenorfine). PBS-injected mice were used as control group for all experimental conditions to reduce animal numbers. Mice were excluded from further analysis in case of irregularities during surgery that could affect infarct size including lack of blanching of the cardiac tissue after ligation or when the location of the suture was incorrect. Number of animals per experiment is shown in figure legends.

### TTC Staining

After 2 days, the animals received a lethal dose of anesthesia, after which the hearts were flushed with Evans-blue to identify the transfused area. Subsequently, the hearts were collected, sliced into 1 mm slices, and stained with 1% TTC in Sorensen buffer. Slices were imaged with AxioCamICc3 and infarct size was analyzed with ImageJ (v1.50f). Infarct size was analyzed by measuring total left ventricular area and infarcted area and calculating the infarct as a percentage of total left ventricular area. Analyses were performed blinded.

### Immunostaining

Hearts destined for immunohistochemical analysis were incubated overnight in 15% sucrose solution, embedded in optimal cutting temperature (OCT) compound, and stored at − 80 °C. Cryosections of 7 μm were cut transversally from apex to the site of ligation, with 280 μm intervals after every 33 sections. Sections were dried and fixed with 4% paraformaldehyde before staining. The following antibodies were used for staining the sections: α-Ki67 (rabbit, AB9260, Millipore), α-MF20 (mouse, DSHB), α-αSMA (mouse, A2547, Sigma), α-cTNI (goat, 4 T21/2, HyTest), α-CD31 (goat, SC-1506, Santa Cruz), α-YAP (rabbit, 4912, Cell Signaling), and α-Endoglin (goat, AF1097, R&D systems). The α-YAP antibody was amplified using TSA amplification (Thermo Fisher). After incubation with the appropriate secondary antibody, the sections were counterstained with DAPI and mounted with Fluoromount (Sigma). The sections were imaged using a Leica microscope and scanned with Pannoramic Slide Scanner (3D Histech). Analysis was performed with ImageJ software.

### Quantification of Proliferative Markers

To determine where in the heart EVs induce proliferation markers, the total number of Ki67 and YAP-expressing cells was determined in sections at levels 3 until 8 of a pilot group (see Fig. 3a). The total number of positive nuclei was counted in the infarct area plus border zone, corrected for infarct size. Since levels 5 and 6 showed the highest uptake of EVs and numbers of positive cells, quantification of proliferating cell types in sections of levels 5 and 6 was performed in all the hearts. An area of 1.2 mm^2^ in the border zone was analyzed, spanning the entire width of the ventricle. Cells with Ki67-positive nuclei were categorized based on co-staining with cell type–specific markers and morphology into cardiomyocytes, endothelial cells, and other/interstitial cells. Endoglin was analyzed as total area of endoglin signal in the sections corrected for total cardiac area. All analyses were performed blinded for treatment.

### Statistical Analysis

All data are presented as mean ± SEM. The statistical difference between two groups was analyzed using an unpaired Student’s *t* test, with Welch’s correction in case of unequal variances. For three or more groups, one-way ANOVA was used, with Bonferroni as post hoc test. Level of significance was set at *p* < 0.05.

## Results

### Infarct Size Is Not Affected After Injection of hCPCs with Reduced Vesicle Secretion

**Fig. 1:**
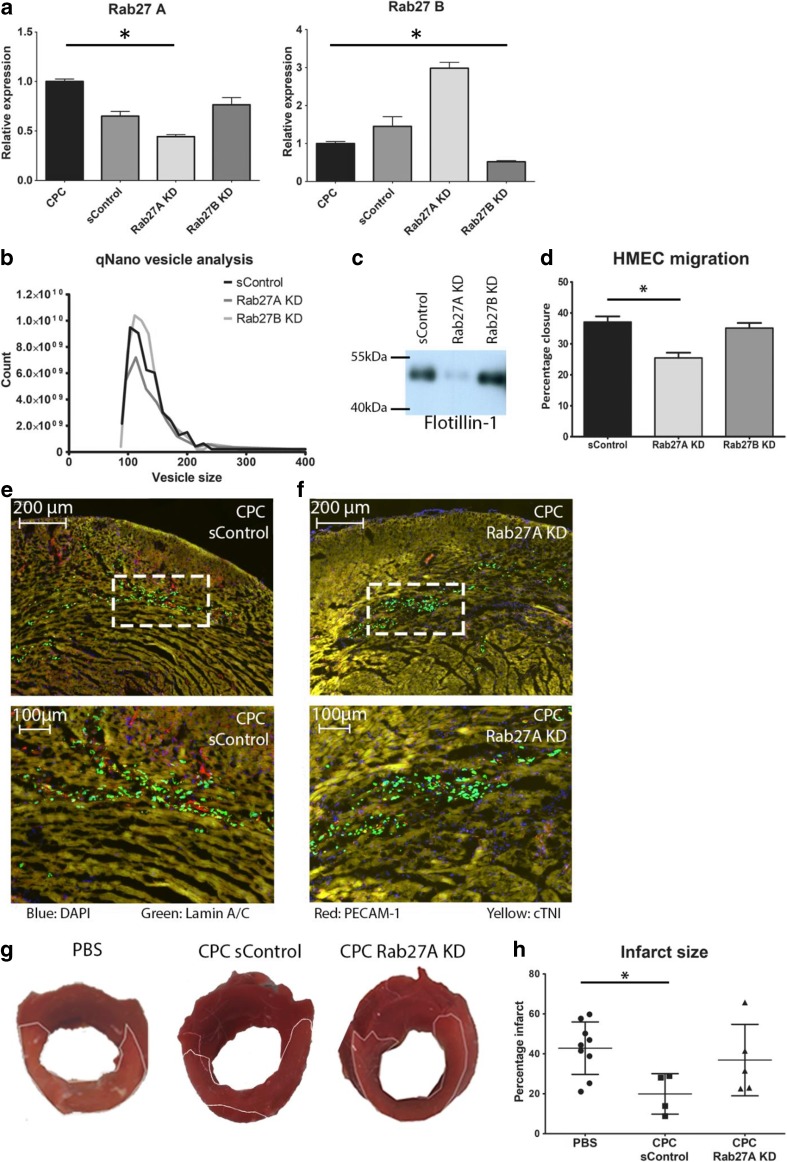
Rab knockdown reduces the secretion of EVs as well as their positive effect in vitro and in vivo. **a** Rab knockdown analysis by qPCR. Both Rab27A and Rab27B are effectively knocked down after lentiviral shRNA transduction. **b** qNano analysis of vesicles in the EV isolate by sControl-hCPCs, and hCPCs with Rab27A and Rab27B knockdown. Only Rab27A KD reduces the number of EVs compared to sControl-hCPCs. **c** Western blot for flotillin-1 confirms reduction of EVs by Rab27A knockdown, while no effect is seen with Rab27B knockdown. **d** Effect of EV isolate on migration of endothelial cells after scratch assay. EV isolate from Rab27A KD has reduced capacity to induce migration compared to sControl-hCPCs EV isolate (EV isolates of equal cell numbers). **e**, **f** Both sControl-hCPCs (**e**) and Rab27 KD-hCPCs (**f**) are observed in the heart after 48 h (blue: DAPI, green: human Lamin A/C, red: PECAM-1, yellow: cardiac troponin I (cTNI)). **g** Representative pictures of TTC analysis after injection of PBS, sControl-hCPCs, and Rab27A KD-hCPCs. **h** Quantification of the infarcted area in hearts injected with PBS, sControl-hCPCs, and Rab27A-KD hCPCs (PBS, *n* = 9; control-hCPC, *n* = 4; Rab27A KD-hCPC, *n* = 5)

Transplantation of hCPC resulted in beneficial effects on the heart after MI [4]. Interestingly, EVs secreted by hCPCs were shown to possess pro-angiogenic activity [7]; hence, paracrine factors may represent an important component of the beneficial effects of cardiac progenitor cell transplantation on the heart after MI. Therefore, our first aim was to examine to what extend the secretion of EVs by hCPCs contributes to this effect. Rab27A and Rab27B are part of the EV secretion pathway, and loss of these proteins has been documented to affect EV secretion [29]. To this end, we transduced hCPCs with lentiviruses expressing shRNA for Rab27A and Rab27B or a scrambled control (sControl). qPCR analysis revealed efficient knockdown of Rab27A and Rab27B (Fig. 1a). To determine if the knockdown influenced the EV secretion of hCPCs, conditioned medium of sControl-hCPCs, Rab27A KD-hCPCs, and Rab27B KD-hCPCs was processed for EV isolation by ultracentrifugation. EV-isolates were resuspended in equal volumes and corrected for the number of cells the conditioned medium was derived from. We confirmed the presence of extracellular vesicles in the conditioned medium of sControl-hCPCs with Western blot for EV markers and by determining the density of vesicles (Supplemental Fig. 1A, B). qNano technology revealed a population of vesicles within a size range of 50–150 nm, with a mean diameter ± 100 nm (Supplemental Fig. 1C), suggesting the presence of exosome-like vesicles, which could be confirmed by EM (Supplemental Fig. 1D) [10, 27]. In the EV-isolates of Rab27A KD-hCPCs, the number of secreted vesicles was decreased, while Rab27B knockdown did not affect vesicle secretion (Fig. 1b). Although Rab27A knockdown increased the mRNA levels of Rab27B, this did not restore the number of secreted EVs to sControl-hCPC levels (Fig. 1a, b). Western blot analysis for exosomal-enriched protein Flot-1 confirmed a drastic reduction of the amount of EVs in the EV isolates upon Rab27A knockdown, while no difference was seen in the EV isolates from Rab27B KD-hCPCs compared to sControl-hCPCs (Fig. 1c**)**. We have previously reported that migration of endothelial cells was increased after stimulation with EVs from hCPCs [7]. Therefore, we examined whether migration of HMEC-1 cells was attenuated when subjected to EV-isolates from the Rab27A KD-hCPCs, compared the sControl-hCPCs. Indeed, isolations from Rab27A KD-hCPCs were unable to stimulate the migration of HMEC-1 cells in a scratch assay to the same extend as sControl-hCPC (Fig. 1d).

To determine if the EV secretion is responsible for the cardioprotective effect of hCPCs after myocardial infarction, we injected either sControl-hCPCs or Rab27A KD-hCPCs into the infarct border zone after induction of MI. Immunohistological analysis after 48 h showed the presence of both sControl-hCPCs (Fig. 1e) and Rab27A KD-hCPCs (Fig. 1f) in the heart. To determine the effect on infarct size, we performed a TTC staining and observed that sControl-hCPCs significantly reduced the infarct size when compared to PBS injections (Fig. 1g, h). This effect was not present upon injection of Rab27A KD-hCPCs, suggesting that the vesicles secreted by hCPCs are a major contributor to the reduction of infarct size by hCPCs.

### hCPC-Derived EVs Reduce Infarct Size

**Fig. 2:**
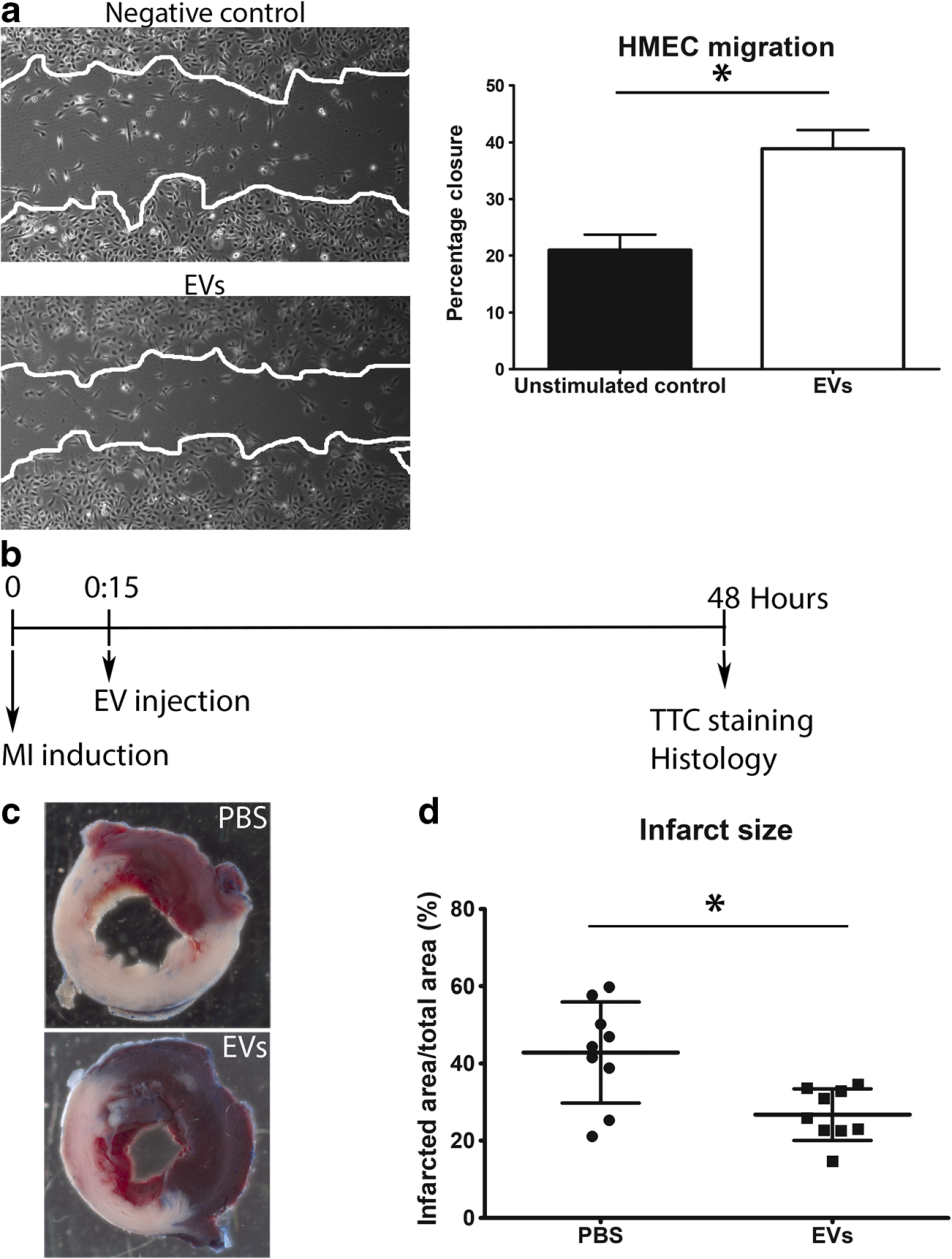
hCPC-derived EVs reduce infarct size. **a** EVs stimulate wound closure in a HMEC-1 scratch assay. Percentage of closure of the wounded area was determined after 24 h. **b** Timeline of in vivo procedures. **c** Representative images of TTC stained hearts injected with PBS or exosomes, 48 h after MI. White area represents the infarcted region. **d** Quantification of the infarcted area as a percentage of the total area of the left ventricle (*n* = 9)

Since Rab27A KD-hCPCs did not reduce infarct size, suggesting a role for the extracellular vesicles, we next sought to directly analyze the effect of hCPC-derived EVs upon injection into the infarcted myocardium. Prior to injecting the EVs, we confirmed the presence of a population of EVs of ± 120 nm in size with a density of approximately 1.12 g/mL (data not shown). Furthermore, functional activity of EVs was evaluated by performing a scratch assay, which revealed that these EVs stimulated the migration of HMEC-1, as we previously reported [6] (Fig. 2a**)**. EVs were subsequently injected into the border zone of the infarction, 15 min after permanent ligation of the LAD, and the infarct size was analyzed after 48 h (Fig. 2b). As shown by TTC analysis (Fig. 2c), PBS-injected hearts displayed a mean infarct size of 42.9% ± 4.4, while the infarct size in hearts injected with EVs was 26.75% ± 2.2 (Fig. 2d), indicating that hCPC-EVs reduce the extent of damage shortly after MI.

### EV Uptake by Cardiovascular Cells Post-MI in the Myocardium

**Fig. 3:**
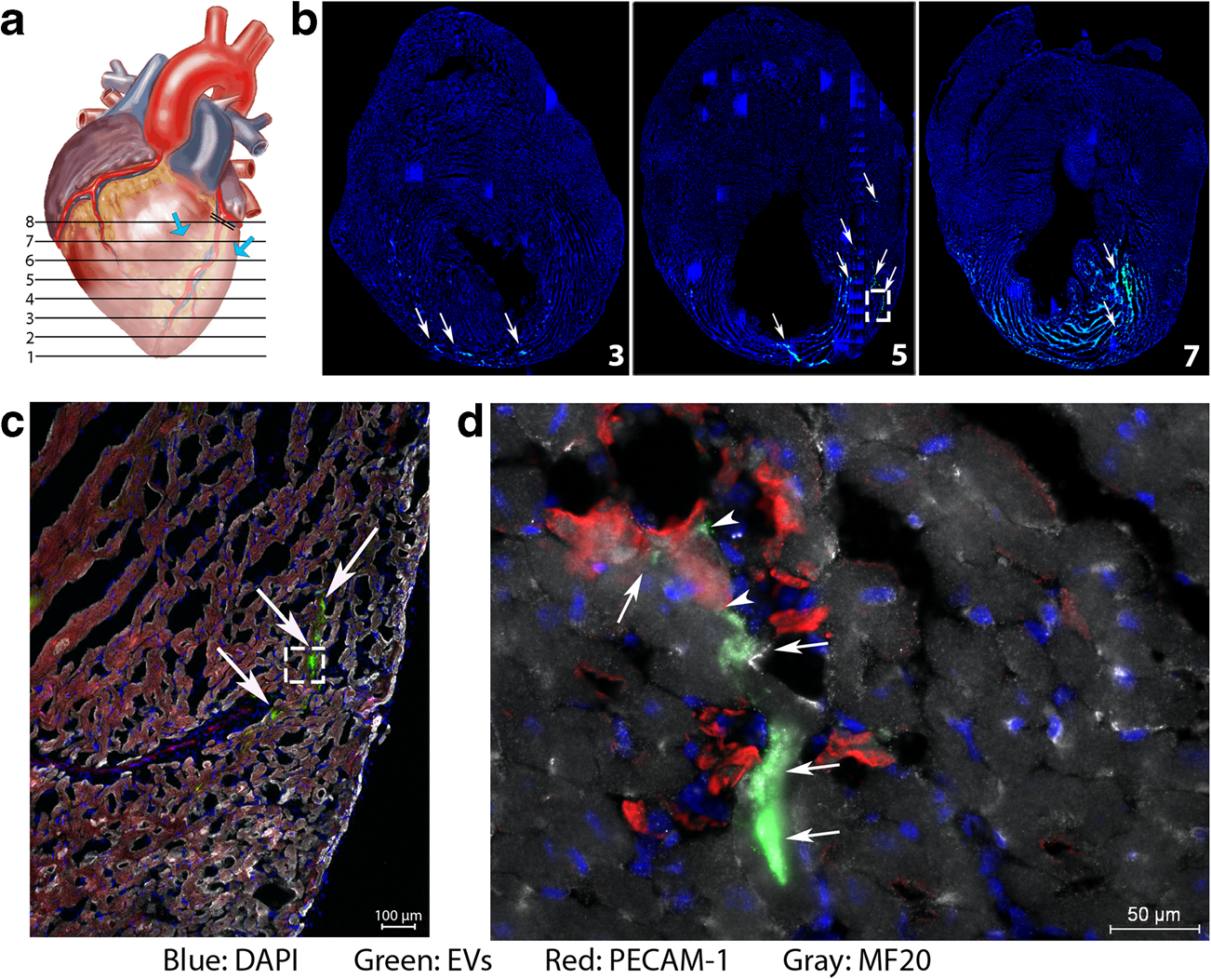
EV distribution in the heart. **a** Schematic representation of the analysis of infarcted heart. Blue arrows indicate the EV injection site. **b** Analysis of the entire heart showed the uptake of PKH67 from the loaded EVs in the heart from level 3 to 7/8. White arrows point at positive cells. **c** Higher magnification of insert in B, showing uptake of EVs in the infarct zone. **d** Higher magnification of insert in C, which shows uptake of EVs by cardiomyocytes (arrows) and endothelial cells (arrowheads)

To understand how EVs could exert their effect shortly after MI, we determined which of the cardiovascular cells present within the injured myocardium internalize EVs. Therefore, we injected EVs labeled with PKH67 or PKH26 into mouse hearts that underwent MI and analyzed the distribution of the fluorescently labeled EVs in combination with cell type–specific immunofluorescent analysis (Fig. 3a). After EV labeling, sucrose gradient separation was used to prevent free label injections into the myocardium. As can be appreciated, EV fluorescence was spread from the site of injection towards the apex, showing a large area of EV uptake from levels 3 to 8 (Fig. 3b). The total area of EV dye uptake extended to over 2700 μm. Most EVs were taken up by cells in the border zone, close to the anticipated sites of injection (levels 5 and 6). To determine which cell types contained EVs, we analyzed high-magnification images (Fig. 3c). The dominant target cells for hCPC-derived EVs were endothelial cells and cardiomyocytes, although some interstitial cells also seemed to be positive for EV uptake (Fig. 3d, single channel pictures in Supplemental Fig. 2). Thus, EVs are effectively taken up by cardiomyocytes and endothelial cells after injection into the ischemic left ventricle.

### EVs Increased the Number of Ki67- and YAP-Expressing Cells in the Left Ventricle

**Fig. 4:**
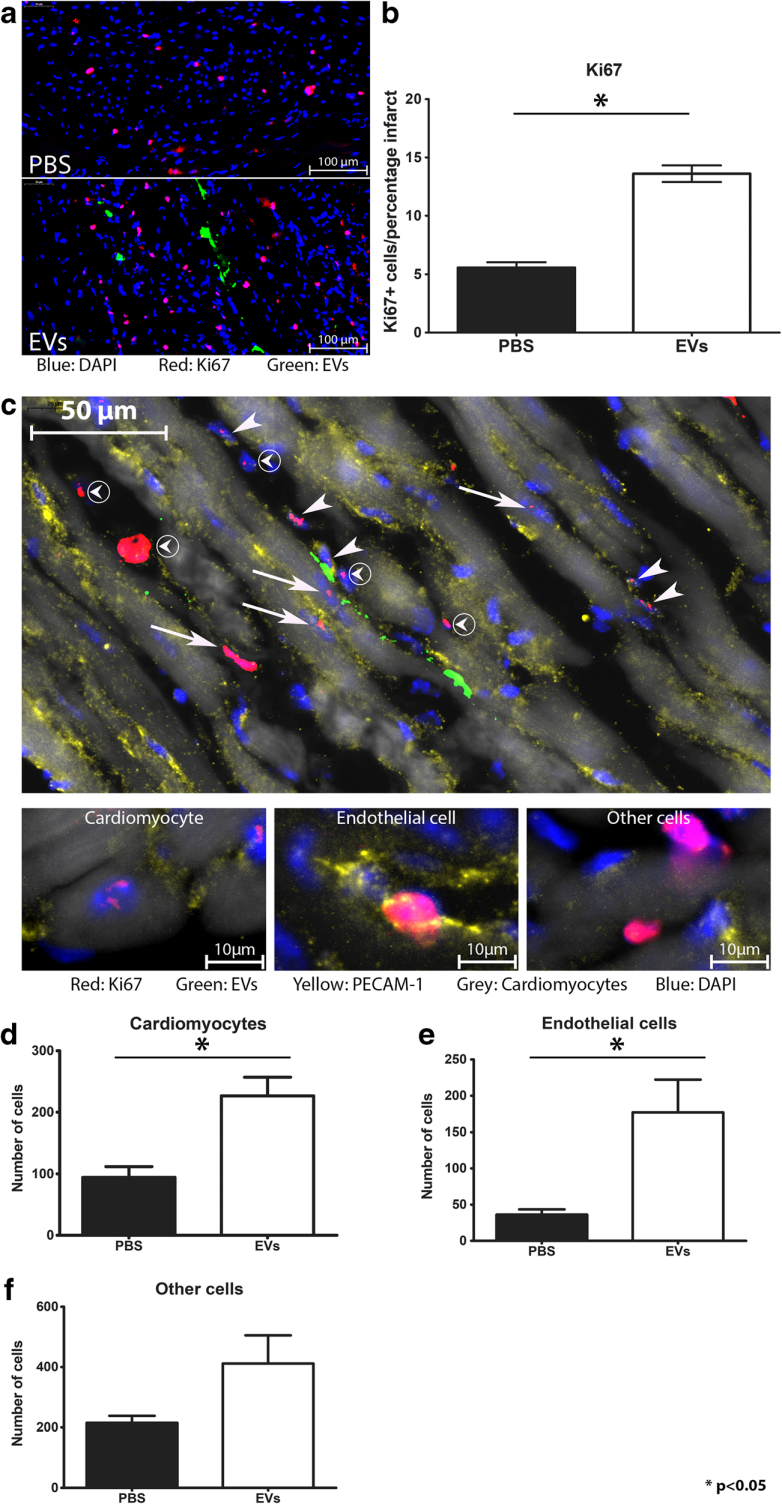
hCPC-derived EVs induce Ki67 expression in the left ventricle after MI. **a** Ki67 (red) in the heart in the presence of EVs (green). Nuclei are stained with DAPI (blue). **b** Quantification of Ki67-positive cells in the infarct and border zone, relative to the area of the infarct, shows that EVs increase the number of Ki67-positive cells (*n* = 3). **c** Ki67 staining in the heart after EV injection shows proliferation in various cell types (arrows: positive cardiomyocytes; arrowheads: positive endothelial cells; circled arrowheads: other cells). Smaller pictures show examples of individual cell types. Cardiomyocytes were identified through autofluorescence. **d**–**f** Graphs depicting the number of proliferating cardiomyocytes (**d**), endothelial cells (**e**), and other cells (**f**) in the heart, in layers 5 and 6, after EV and PBS injection. A significant increase was found in cardiomyocyte proliferation (*n* = 3)

Since EV injection reduced infarct size, we analyzed the EV-positive area in more detail. We first assessed proliferation of cells within the infarcted area by determining the number of Ki67-positive cells. Interestingly, we found Ki67 expression not only within the PKH-positive cells but also more present in cells in the vicinity of the EV signal (Fig. 4a). Quantification of these cells, by counting the number of positive nuclei in relation to the infarct area, showed a significant increase in the number of Ki67-positive nuclei in the hearts receiving hCPC-derived EVs compared to PBS-injected heart (Fig. 4b). Number of positive cells was corrected for infarct size in order to correct for differences in cardiac size. Next, we analyzed which of the cell types found in the border zone of the infarct were Ki67 positive. As shown in Fig. 4c, we found Ki67-positive nuclei in cardiomyocytes, endothelial cells, and other/interstitial cells, which is in agreement with the cell types we identified before. Since the largest increase in proliferation was seen in layers 5 and 6 (Supplemental Fig. 3), we quantified the specific number of different cell types in the border zone of the infarct in these levels. As can be seen in Fig. 4d, hCPC-EV injection resulted in a significant increase in Ki67-positive cardiomyocytes compared to PBS control (mean ± st.dev, 226.5 ± 74.31 vs 94.17 ± 43.20, *p* < 0.05). A similar effect was seen for endothelial cells, where EV injection increased the number of Ki67-positive cells from 36.17 ± 17.88 to 177.0 ± 110.9 (*p* < 0.05) (Fig. 4e). The difference in Ki67-positive cells in other cell types did not reach statistical significance (PBS, 215.2 ± 56.53 vs EV, 411.7 ± 228.4) (Fig. 4f).

**Fig. 5:**
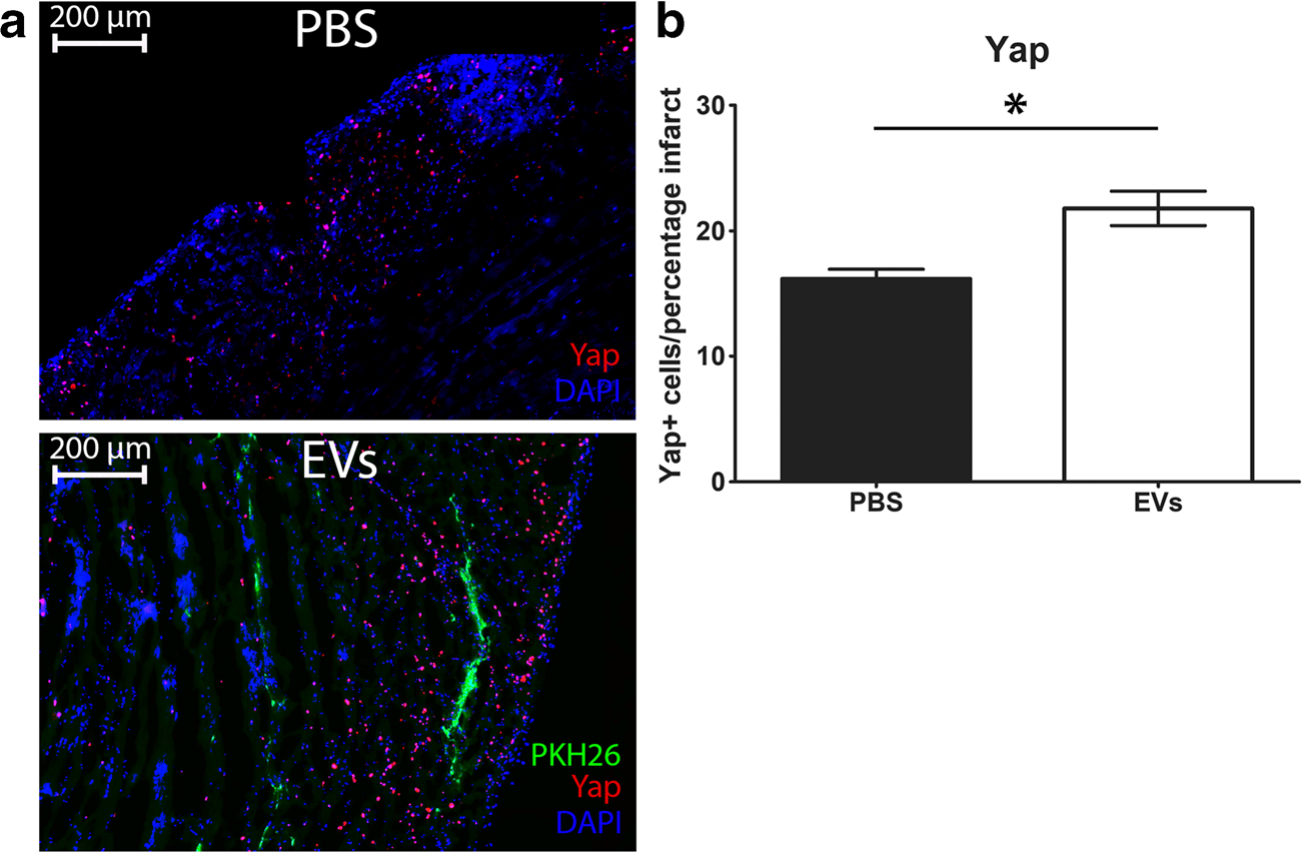
YAP expression after MI. **a** Representative pictures of YAP expression in the border zone of PBS and EV injected hearts. **b** Quantitative analysis shows that the number of YAP-positive cells is higher in the infarct zone of hearts injected with hCPC-EVs compared to PBS-injected hearts. Numbers of positive cells are normalized for infarct size and shown as number of cells per percentage infarct

In order to investigate how EVs can increase Ki67 after MI, we looked into the association with activation of YAP. In tumor and progenitor cells, it is shown that Ki67 correlates to YAP expression and furthermore, a decrease in YAP causes a decrease in Ki67 levels and a decrease in proliferation [30–32]. Therefore, we explored whether EV treatment affects YAP levels. Interestingly, we observed an increase in nuclear YAP in the border zone in the vicinity of the EV signal (Fig. 5a, single channels Supplemental Fig. 4**)**, and a significant increase of total YAP in the infarcted area (Fig. 5b). In summary, injection of hCPC-EVs increases Ki67 and YAP signaling in the heart, of which the former is predominantly increased in cardiomyocytes and endothelial cells.

### Endoglin Level in the Heart Is Influenced by EVs

**Fig. 6:**
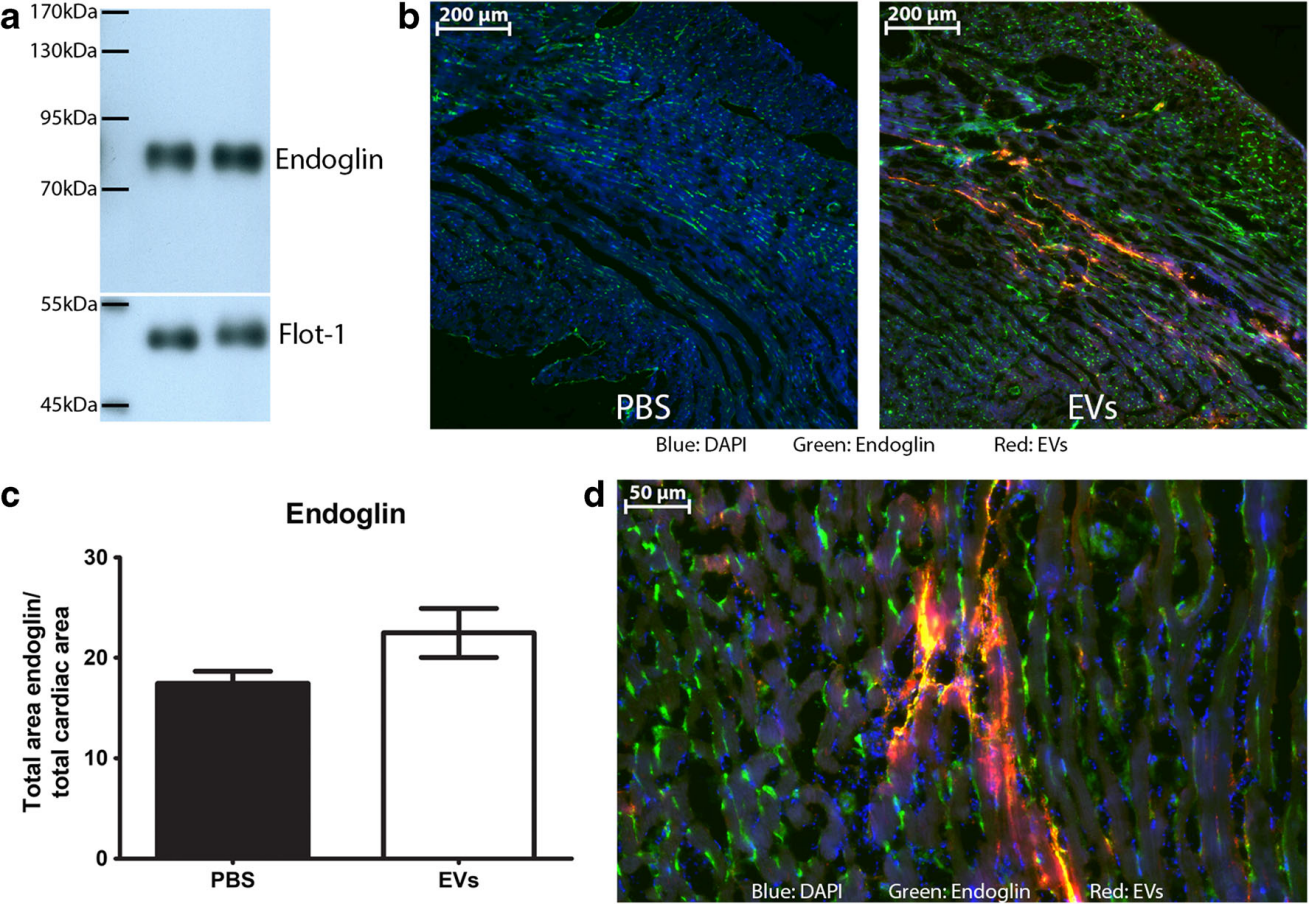
Endoglin levels in the heart. **a** Endoglin is present in EVs from hCPCs, two individual isolations are shown. **b** After injection of PKH-labeled EVs, areas positive for EV uptake are also highly positive for endoglin, compared to PBS injection. **c** Quantification of endoglin levels in the entire heart after PBS or EV injection. **d** Endoglin signal is mainly found in endothelial cells and cells positive for EV uptake

Since we observed an increase in endothelial cell proliferation, we next questioned whether hCPC-EV treatment affected endothelial activation. We have previously shown that EVs from hCPCs are a very potent stimulant of angiogenesis [7]. Since changes in vascular density are not yet measurable at this short-time point post-MI, we turned to endoglin as a well-known factor in inducing angiogenesis and a marker of active endothelial cells [33] and determined whether endoglin expression was changed after hCPC-EV injection. As shown in Fig. 6a, endoglin is clearly present in hCPC-EVs, confirming their pro-angiogenic profile. Next, we analyzed if endoglin expression was increased in the injured myocardium after hCPC-EV injection. We observed an increase in endoglin expression in the infarct border zone in the area of EV uptake, when compared to control (Fig. 6b, c). Higher magnification showed mainly endothelial cells and small vessels with a strong endoglin signal. Endoglin staining also partially colocalized with EV uptake, suggesting delivery of endoglin by EVs (Fig. 6d). Although the difference in total endoglin levels did not reach significance, it does suggest that EVs, through delivery of endoglin itself and other pro-angiogenic stimuli, cause an increase in endoglin levels.

## Discussion

Cell transplantation has been extensively studied over the last decade as a regenerative therapy for the heart. The transplantation of hCPCs has been shown to increase cardiac function, induce neovascularization and, in some cases, provide new cardiomyocytes [3, 4, 34]. However, in the last few years, it has become clear that this beneficial effect on heart performance is mainly due to the paracrine factors secreted by these cells [35]. In this study, we show that EVs from hCPCs can decrease infarct size by inducing proliferative markers and by altering the angiogenic state of the tissue, as shown by the presence of endoglin positive–activated blood vessels.

Several studies have shown that injection of hCPCs exerts beneficial effects after MI and that hCPCs are able to differentiate to cardiomyocytes and endothelial cells in the mouse heart [3, 4, 34]. Even as soon as 2 days post-MI, when hCPC differentiation is still absent, a positive effect is already seen, suggesting the effect of paracrine factors [5]. Therefore, we set out to discern between the effect of hCPCs and their secreted EVs on short-term cardiac regeneration and infarct size. Knockdown of Rab27A in hCPCs, previously shown to affect EV secretion in tumor cells [29], also reduced the number of EVs secreted from hCPCs. The observed increase in Rab27B was not sufficient to rescue the EV secretion, showing that Rab27A is crucial in the secretion pathway in hCPCs. This dependence on Rab27A has also been seen in other cells [36]. The in vitro effect on endothelial cell migration was reduced when stimulated with the EV isolate from Rab27A knockdown cells, confirming a loss of paracrine effects. Injection of the cells into the murine heart after MI showed that normal hCPCs can decrease the infarct size after 2 days, which confirms the effect on cardiac function seen before [4]. In contrast, we could not find a decrease in infarct size with RAB27A KD-hCPCs, showing that on the short term, the paracrine factors are a major contributor to the cardioprotective effect.

It has been shown that paracrine factors from, e.g., mesenchymal stromal cells (MSCs) and cardiac progenitor cells have beneficial effects after MI. Injection of the conditioned medium from MSCs has confirmed this hypothesis by showing a decrease in infarct size and increase in cardiac function [37, 38]. Since hCPCs are part of the cardiac cell lineage, their paracrine factors could be more tailored towards cardiac regeneration. For example, conditioned medium of hCPCs has been shown to induce angiogenesis and migration of hCPCs [39]. Following this, multiple studies have shown that injection of EVs—of several cell sources—is capable of reducing cardiac infarction in mice and rats more than 1 week after MI induction [40–49]. The first large animal experiment using cardiosphere-derived EVs revealed a reduction in infarct size in an acute MI setting; however, it failed to show an improvement in cardiac function in a chronic model [50]. A short-term effect on infarct size, within 48 h, has only been illustrated with EVs from human MSCs [51]. Arslan and colleagues observed that the decrease in infarct size coincided with an increase in pAkt and pGSKβ signaling, known mediators in survival pathways of the cells. Here, we demonstrate that EVs from hCPCs are also capable of reducing the infarct size after 48 h. This is in corroboration with the results on cardiac functions after injection of hCPCs and supports the theory that paracrine factors are a major effector of cell therapy [4, 5].

Proliferation of cardiac cells after MI can prevent the extent of the damage and result in a preservation and regeneration of contractile force. So far, proliferation has only been seen after treatment with ES-derived EVs and hypoxic MSC EVs [45, 52]. Khan et al. have shown an increase in proliferation of resident c-kit positive cells, via phospho-histone 3 staining [45], upon EV injection. Zhu et al. showed that MSC EV-treated hearts had an increase in overall proliferation by Ki67 staining, but could not find a significant increase in cardiomyocyte proliferation [52]. We show here that injection of EVs from hCPCs leads to stimulation of expression of proliferative markers in the heart after MI and that this effect is already present after 48 h. After EV injection, an overall increase in proliferation could be observed in the infarcted and border zone area of the hearts. In particular around the area of EV uptake, the increase in proliferation, shown by Ki67 staining, was striking. Moreover, detailed analysis showed a specific increase in cardiomyocyte and endothelial cell proliferation, suggesting that these cell types are mainly being affected by the injected EVs. Interestingly, Ki67 was shown to correlate with YAP levels and a decrease in YAP expression levels caused a reduction in Ki67 [30–32]. In corroboration with the increase in Ki67 we observe in our EV-injected hearts, we also observed an increase in YAP in the vicinity of EV uptake. This is in line with reports showing that YAP is involved in cell growth [21, 53] and that EVs are able to activate YAP signaling [54, 55]. Interestingly, Yap is part of the Hippo pathway and deficiencies in Hippo signaling have shown to be beneficial in heart failure [19]. Moreover, analysis of miRNAs involved in cardiomyocyte proliferation showed that the majority inhibit the Hippo pathway and lead to nuclear YAP localization [56]. The increase in YAP we see therefore implies a link to proliferation and cardiac regeneration. However, to establish a direct correlation between cardiomyocyte proliferation and hCPC-EVs with regard to YAP signaling, in-depth in vitro experiments will be required*.* Since the Hippo-YAP pathway is also related to angiogenesis [57], and we observe an increase in Ki67-expressing endothelial cells, the increase in YAP after EV treatment could also affect endothelial function and neo-vascularization. Altogether, our results indicate that hCPC-EVs are capable of increasing proliferative markers in the cardiac tissue.

Since we have observed the increase in Ki67 also in endothelial cells, we raised the question whether angiogenesis was also affected by the hCPC-EVs here, since we have shown previously that hCPC-EVs are very potent inducers of angiogenesis [6, 7]. This increase in angiogenesis was seen in vitro as well as in vivo and shown to be dependent on EMMPRIN. Therefore, since we analyzed the effects after 48 h, we investigated the activation of endothelial cells after hCPC-EV injection through endoglin. Endoglin, a co-receptor for the TGF-β/ALK1 signaling pathway, is a known pro-angiogenic factor and is present on activated endothelial cells [33, 58]. We found that endoglin is present on the hCPC-EVs and that the endoglin signal was increased in and around the area of hCPC-EV uptake after hCPC-EV injection. This signal was mainly seen in endothelial cells and small vessels, suggesting primarily endothelial activation of the smaller capillaries. Although the quantification did not reach statistical significance, probably due to the already activated post-MI responses, the observation of the increased endoglin signal indicates more local and small vessel activation. This suggests that hCPC-EVs can activate endoglin in the cardiac cells and could thereby increase the activation of endothelial cells.

The intricacy of their content, consisting of several (mi)RNAs and proteins, and the effectiveness of EVs make them interesting potential therapies. Their ability to convey several signals and to be taken up by virtually any cell is an indispensable quality for an effective regenerative therapy, and makes them very suitable as an off-the-shelf treatment. We show that hCPC-secreted EVs likely contribute to the reduced cardiac deterioration observed in pre-clinical cell transplantation studies. They increase proliferation in the left ventricle and promote cardiomyocyte proliferative markers in the border zone. Furthermore, they can influence angiogenesis by stimulation of pro-angiogenic factors such as endoglin. Further research into the mechanisms by which the EVs exert this effect would provide better insight into the therapeutic range of the EVs. Altogether, hCPC-EVs exert cardioprotective effects shortly after MI, making them promising novel therapeutic agents.

## Electronic Supplementary Material

ESM 1 (DOCX 702 kb)

## Abbreviations

EGF: Epidermal growth factor
EMMPRIN: Extracellular matrix metalloproteinase inducer
EV: Extracellular vesicles
hCPCs: Human cardiac progenitor cells
MSC: Mesenchymal stromal cells
MI: Myocardial infarction
OCT: Optimal cutting temperature compound
PBS: Phosphate-buffered saline
Rab27A knock down: Rab27A KD
sControl: Scrambled control
YAP: Yes-associated protein
