# Supplementary material for: Cardiac Progenitor Cell–Derived Extracellular Vesicles Reduce Infarct Size and Associate with Increased Cardiovascular Cell Proliferation
Source: J Cardiovasc Transl Res. 2018 Nov 19;12(1):5–17. doi: 10.1007/s12265-018-9842-9 (PMC6394631; doi:10.1007/s12265-018-9842-9)
Supplement: Supplementary file 1 — (DOCX 702 kb) [file 12265_2018_9842_MOESM1_ESM.docx]

Supplementary Material

Cardiac Progenitor Cell-Derived Extracellular Vesicles Reduce Infarct Size and Associate with Increased Cardiovascular Cell Proliferation

Janita A. Maring^1^, Kirsten Lodder^1^, Emma Mol^2^, Vera Verhage^2^, Karien .C. Wiesmeijer^1^, Calinda K.E. Dingenouts^1^, Asja T. Moerkamp^1^, Janine C. Deddens^2^, Pieter Vader^2,3^, Anke M. Smits^1^, Joost P.G. Sluijter^2,4^, Marie-José Goumans^*1^

*** Correspondence:** Marie-José Goumans: m.j.goumans@lumc.nl

# Supplementary Figures and Tables


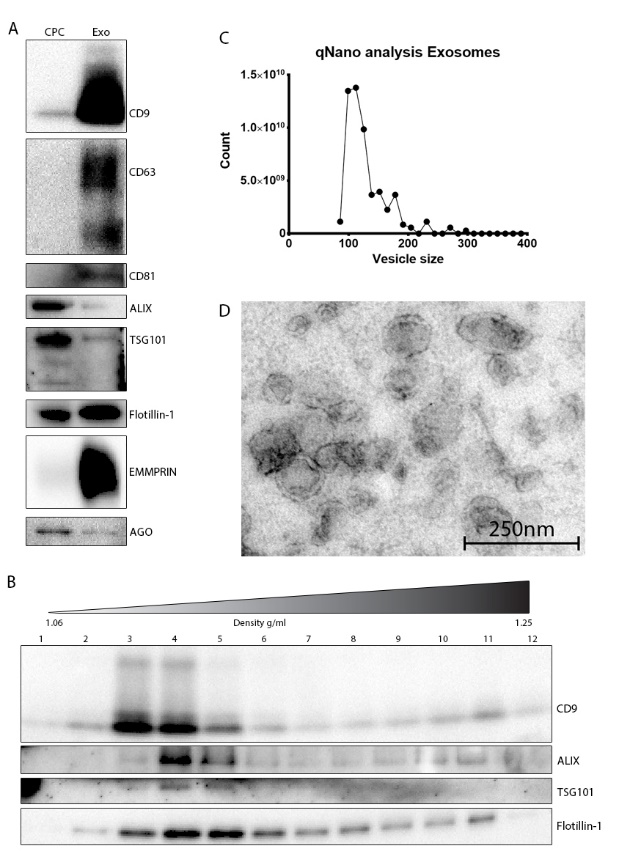


Figure S1: Quality control of hCPC EVs. (A) Western Blot for known EV and exosomal markers, and AGO, which is underrepresented in vesicles. (B) EV isolation by sucrose gradient was collected in 12 different fractions that were individually analysed for the presence of EV. Western blot analysis is shown for CD9, ALIX, TSG101 and Flotillin-1. The triangle above the Western Blot represents the density of the numbered fractions, ranging from 1.06 g/ml (left, fraction 1) to 1.25 g/ml (right, fraction 12). EVs are specifically present at 1.12 g/ml. (C) qNano analysis of vesicles secreted by hCPCs. The main population of vesicles is found at 120nm. (D) TEM picture of vesicles isolated from hCPCs, magnification at 43000x.


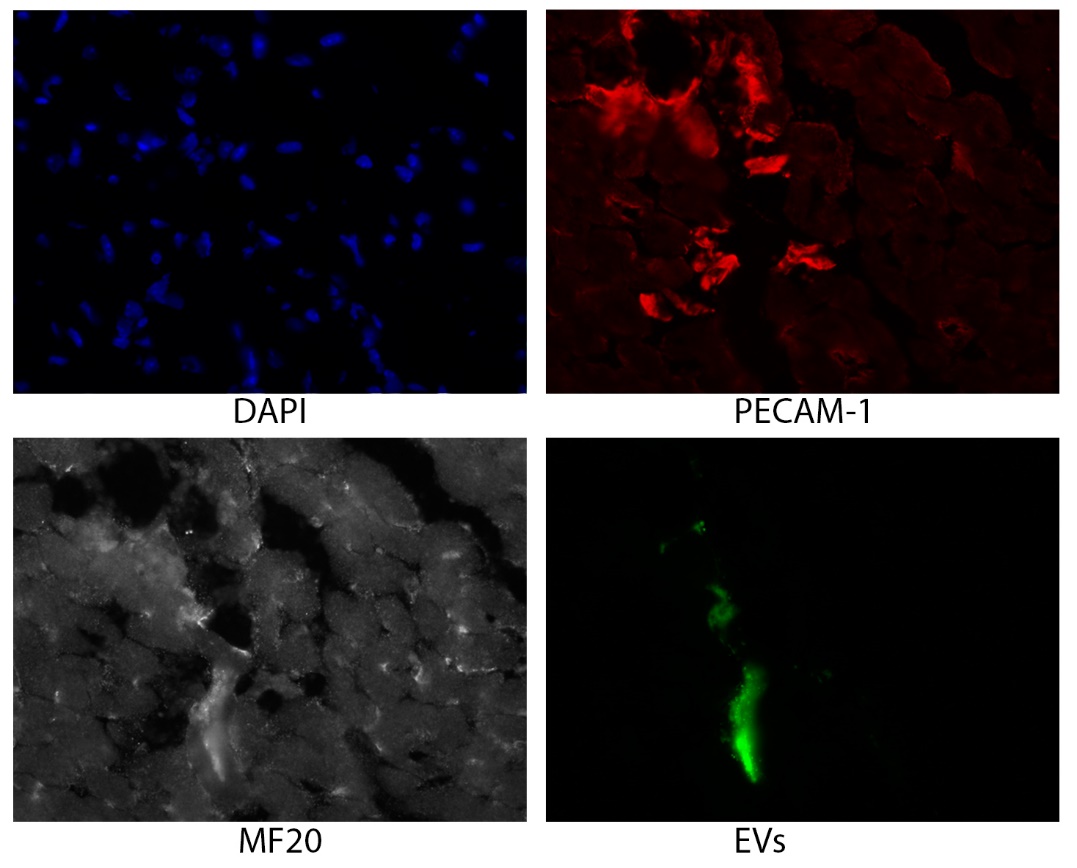


**Figure S2: EV uptake by cardiomyocytes and endothelial cells.** Single channel views of Figure 3D, showing the distribution of the EV signal (PKH67) compared to stainings for DAPI, endothelial cells (PECAM-1) and cardiomyocytes (MF20).


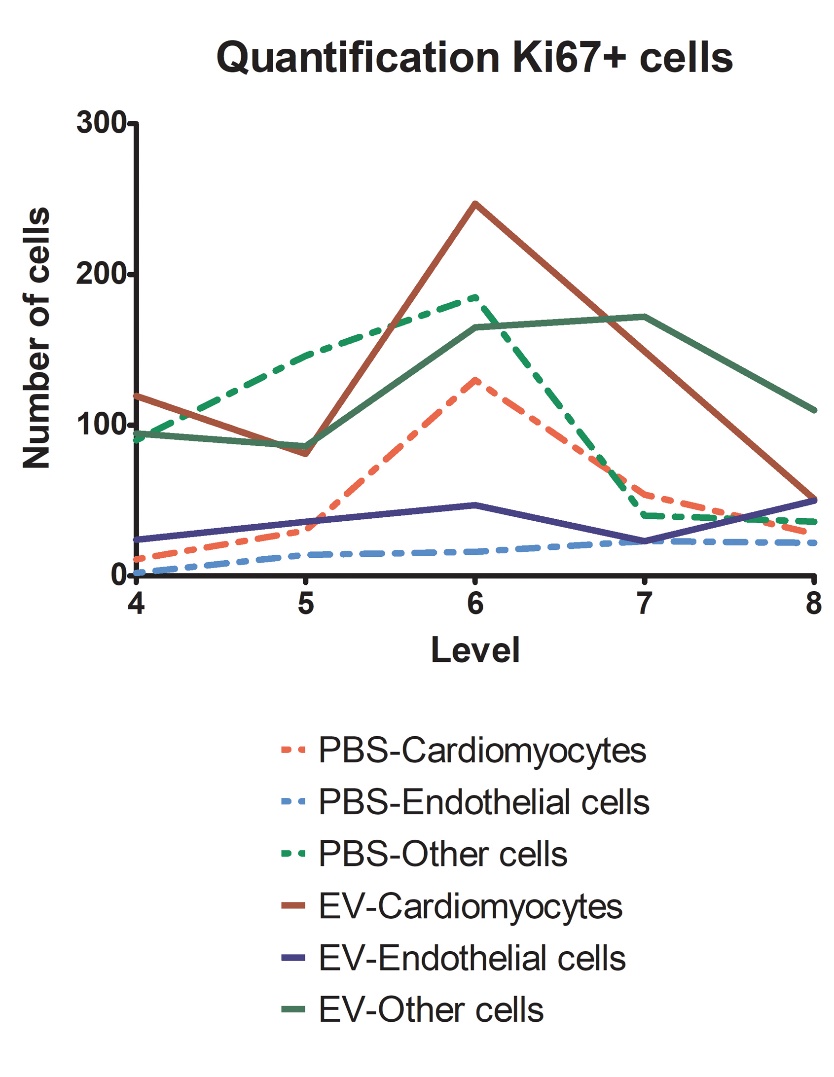


**Figure S3: Distribution of proliferation.** Analysis of proliferating cardiomyocytes, endothelial cells and other/interstitial cells from level 4 through 8 in the heart. Increase in proliferation starts at level 5 and peaks at level 6.


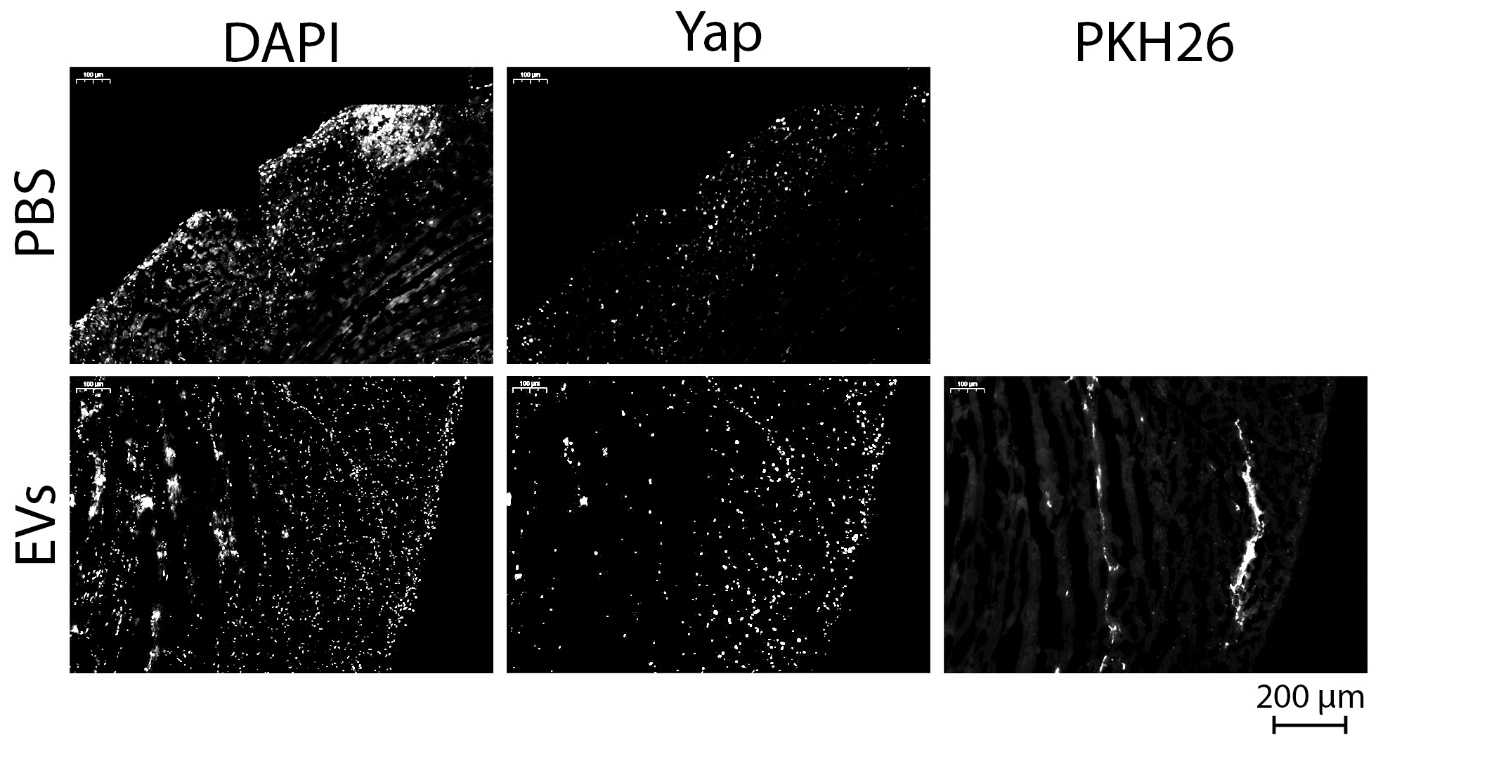


**Figure S4: Yap signal after EV injection.** Single channel representation of Figure 5A, showing the individual channels for DAPI, YAP and PKH26 (EVs).
